# In Silico Design of Dual Estrogen Receptor and Hsp90 Inhibitors for ER-Positive Breast Cancer Through a Mixed Ligand/Structure-Based Approach

**DOI:** 10.3390/molecules29246040

**Published:** 2024-12-21

**Authors:** Gabriele La Monica, Federica Alamia, Alessia Bono, Francesco Mingoia, Annamaria Martorana, Antonino Lauria

**Affiliations:** 1 Dipartimento di Scienze e Tecnologie Biologiche Chimiche e Farmaceutiche “STEBICEF”, University of Palermo, Viale delle Scienze, Ed. 17, 90128 Palermo, Italy; gabriele.lamonica01@unipa.it (G.L.M.); federica.alamia01@unipa.it (F.A.); alessia.bono01@unipa.it (A.B.); annamaria.martorana@unipa.it (A.M.); 2 Istituto per lo Studio dei Materiali Nanostrutturati, Consiglio Nazionale delle Ricerche (CNR), 90128 Palermo, Italy; francesco.mingoia@ismn.cnr.it; 3 NBFC—National Biodiversity Future Center, Piazza Marina 61, 90133 Palermo, Italy

**Keywords:** estrogen receptor (ER), Hsp90 inhibition, endocrine resistance, multitarget drug design, DRUDIT, NCI database, breast cancer

## Abstract

Breast cancer remains one of the most prevalent and lethal malignancies in women, particularly the estrogen receptor-positive (ER+) subtype, which accounts for approximately 70% of cases. Traditional endocrine therapies, including aromatase inhibitors, selective estrogen receptor degraders/antagonists (SERDs), and selective estrogen receptor modulators (SERMs), have improved outcomes for metastatic ER+ breast cancer. However, resistance to these agents presents a significant challenge. This study explores a novel therapeutic strategy involving the simultaneous inhibition of the estrogen receptor (ER) and the chaperone protein Hsp90, which is crucial for the stabilization of various oncoproteins, including ER itself. We employed a hybrid, hierarchical in silico virtual screening approach to identify new dual ER/Hsp90 inhibitors, utilizing the Biotarget Predictor Tool (BPT) for efficient multitarget screening of a large compound library. Subsequent structure-based studies, including molecular docking analyses, were conducted to further evaluate the interaction of the top candidates with both ER and Hsp90. Supporting this, molecular dynamics simulations demonstrate the high stability of the multitarget inhibitor **755435** in complex with ER and Hsp90. Our findings suggest that several small molecules, particularly compound **755435**, exhibit promising potential as dual inhibitors, representing a new avenue to overcome resistance in ER+ breast cancer.

## 1. Introduction

Breast cancer is the most common malignancy in women and remains one of the leading causes of cancer-related mortality [1,2]. Based on the expression profiles of estrogen (ER) and progesterone (PR) receptors, as well as and human epidermal growth factor receptor 2 (HER2), this tumor is classified into different subtypes: luminal A, luminal B, HER2+, and basal. The estrogen receptor-positive (ER+) is the most common, accounting for around 70% of diagnoses, both in premenopausal (60%) and postmenopausal women (75%) [3,4,5].

The estrogen receptor (ER) is a nuclear transcription factor that regulates the growth and proliferation of breast cancer cells. It occurs mainly in two isoforms, ERα and ERβ, with the former playing a dominant role in the pathogenesis of breast cancer [6,7].

Over the past 30 years, endocrine therapy for the treatment of ER+ metastatic breast cancer (MBC) has generally included aromatase inhibitors (AIs, oral anastrozole, letrozole, and exemestane), selective estrogen receptor degraders/antagonists (SERDs, intramuscular fulvestrant), and selective estrogen receptor modulators (SERMs, oral tamoxifen) [8]. Tamoxifen, the first approved SERM, is now widely used in both adjuvant and metastatic breast cancer therapy [9,10]. However, evidence of the greater efficacy of AIs and the side-effect profile of tamoxifen have led to the development of new SERM-type inhibitors. *Raloxifene*, for example, has shown comparable efficacy to tamoxifen in the prevention of breast cancer in high-risk women without increasing the risk of endometrial cancer [9].

Despite these advancements, resistance to endocrine agents remains a significant challenge in the treatment of ER+ breast cancer [7,11]. Nonetheless, by combining ER inhibition with other molecular targets, it is possible to disrupt several crucial signaling pathways involved in tumor survival and growth, thereby reducing the likelihood of cancer cells developing resistance [12,13,14]. An emerging strategy could be the simultaneous inhibition of the estrogen receptor (ER) and heat shock protein 90 (Hsp90) [15,16,17]. In the genomic signaling pathway, ERs, initially cytosolic and inactive, become active upon binding with estrogens and chaperones such as Hsp70 and Hsp90. This binding induces conformational changes in the ER, leading to dissociation from the chaperones and the formation of dimers. The estrogen–ER complex is then translocated to the nucleus, where it activates gene transcription and facilitates interaction with co-activators or co-repressors [3]. In this context, the development of multitarget drugs emerges as a pivotal strategy to overcome the limitations of monotherapy, particularly in the treatment of endocrine-resistant breast cancer. Multitarget agents have the ability to simultaneously modulate multiple molecular pathways, thereby enhancing therapeutic efficacy by disrupting tumor cell survival mechanisms and mitigating the emergence of drug resistance. Notable examples from the literature include PROTAC degraders targeting ERα and aromatase, which simultaneously inhibit estrogen signaling and estrogen synthesis, offering a promising solution to endocrine resistance. Another innovative approach involves doxorubicin–formaldehyde conjugates, which combine the DNA binding properties of doxorubicin with the targeted cytotoxic effects of formaldehyde. Lastly, ERα degraders with tubulin inhibitory activity demonstrate dual functionality by degrading ERα while inhibiting tubulin polymerization, effectively disrupting both estrogen signaling and tumor cell cycle progression. The development of multitarget compounds aimed at key targets in complex diseases such as endocrine-resistant breast cancer holds significant potential. This approach not only addresses the limitations of traditional therapies but also offers a more effective and sustainable strategy for managing these challenging malignancies [18,19,20]. Hsp90 is a crucial cellular chaperone that stabilizes and regulates the activity of many oncoproteins, including ER itself, PR, HER2, and components of the EGFR pathway [21]. Hsp90 exists in various isoforms, mainly Hsp90α and Hsp90β in the cytosol, and plays a critical role in stabilizing and functionalizing proteins involved in tumor cell proliferation and survival [16,17,22]. In breast cancer, Hsp90 is involved in various aspects of tumorigenesis, including cellular transformation and angiogenesis, chemoresistance, protection from oxidative stress and cell death, as well as tumor cell proliferation, invasion, and immune recognition [21,22,23].

Hsp90 function is mediated by cycles of dimerization and ATP hydrolysis, which influence the conformation and activity of the protein. The inhibition of Hsp90 not only impairs the stability of ER and other oncoproteins but also affects the overall cellular signaling network and leads to the degradation of proteins that are critical for tumor growth [24,25]. Inhibitors targeting the N-terminal domain of Hsp90 disrupt ATPase activity and prevent the N-terminal domain from binding client proteins. This leads to the degradation of oncoproteins and disruption of various cellular processes [23]. Inhibitors that target the Hsp90 binding domain include natural inhibitors, such as geldanamycin and its analogs, but these have limitations including poor solubility, limited activity, and significant side effects [26]. To overcome these drawbacks, attention has shifted to semi-synthetic inhibitors, also known as first-generation inhibitors. Although many of these drugs have reached Phase I clinical trials, none have progressed beyond Phase II. Therefore, significant efforts have been made to improve binding affinity, potency, side effect profiles, and bioavailability. New synthetic small molecules, known as second-generation Hsp90 inhibitors, have been developed, including the following: (1) purine-based inhibitors [27] (PU-3, BIIB021, BIIB028, MPC-3100, and PU-H71); (2) benzamide-based inhibitors [28] (SNX-5422, also known as PF-04929113); (3) resorcinol-based inhibitors [29] (AUY922, STA9090, AT13387, and KW-2478); and (4) various other inhibitors [30,31].

The simultaneous inhibition of ER and Hsp90 with a single molecule represents an advantageous therapeutic approach compared to the exclusive inhibition of ER. This integrated strategy can overcome drug resistance and improve therapeutic efficacy, as the two targets are interconnected: Hsp90 is essential for the stabilization and activation of ER, and the combined blockade of these two factors can lead to more complete inhibition of oncogenic signaling pathways. Therefore, the identification and development of new inhibitors targeting both ER and Hsp90 can represent a promising therapeutic strategy for the treatment of ER+ breast cancer, with the potential to significantly improve clinical outcomes for patients.

In view of these considerations, an innovative approach has been adopted in this work by integrating hybrid and hierarchical virtual in silico screening to identify new ER/Hsp90 inhibitors. The use of the internal Biotarget Predictor Tool (BPT), in Multitarget Mode, was crucial for enabling efficient screening of an extensive database of active, previously optimized molecules. This advanced computational approach was complemented by in-depth studies based on molecular structure, representing a clear example of how in silico modeling can effectively integrate with experimental methodologies to accelerate the discovery of innovative drugs.

## 2. Results and Discussion

### 2.1. Combined Approach of QikProp and SwissADME for NCI Database Cleaning

For the development of the new virtual screening protocol, we selected the National Cancer Institute (NCI) database due to its extensive collection of compounds (more than 40,000 compounds tested against 60 cancer cell lines) with different chemical and biological properties.

The database was prepared in advance using the LigPrep tool from the Schrödinger Maestro Suite at physiological pH (7.3 ≤ pH ≤ 7.5). This process generated all possible tautomers and stereoisomers for each ligand in their lowest energy states in 3D space.Subsequently, the NCI database was cleaned in two consecutive steps to select small molecules with specific parameters and appropriate drug-likeness criteria. Thus, the ligands were processed using the QikProp tool (Schrödinger, Release 2023-3; QikProp, S., LLC: New York, NY, USA, 2023) [32], which predicts the ADME properties (Absorption, Distribution, Metabolism, and Excretion) of drug candidates based on their full 3D molecular structures. QikProp can predict a wide array of pharmaceutically relevant properties, including octanol/water and water/gas logPs, aqueous solubility logS, brain/blood partition coefficient logBB, overall Central Nervous System (CNS) activity, Caco-2 and MDCK cell permeabilities, and logKhsa for human serum albumin binding, thus enabling rapid screening of compound libraries for potential hits with optimal drug-like properties.

In our study, the analysis was focused on two specific parameters: the “Rule of Five”, which indicates the number of violations of Lipinski’s rule, and “#stars”, a consolidated metric that encapsulates all QikProp parameters into a single value. This value indicates the number of property or descriptor values that fall outside the 95% range for known drugs. A higher number of outlying descriptors results in a higher “#stars” value, suggesting that a molecule is less drug-like compared to one with a lower “#stars” value. To identify only drug-like small molecules, we excluded all ligands with non-zero values for both the Rule of Five and #stars, reducing the NCI database to 18,510 compounds (Supplementary Materials, Databases S1 and S2).

Considering that a significant proportion of drug candidates fail in clinical trials due to poor ADME properties, incorporating ADME predictions into the development process can yield lead compounds with satisfactory ADME performance in clinical trials. This approach reduces the amount of wasted time and resources, streamlining the overall development process. To further predict the drug-like nature of compounds under investigation, we used the SwissADME website (http://www.swissadme.ch, [33]). This platform allowed us to compute physicochemical descriptors and predict ADME parameters, pharmacokinetic properties, and the medicinal chemistry friendliness of our screened small molecules. Specifically, we predicted a set of well-established parameters, such as the number of heavy atoms, H-bond acceptors, H-bond donors, rotatable bonds, Rule of Five, Blood–Brain Barrier (BBB) permeability, metabolic reactions, and Human Oral Absorption, which are also shared with QikProp. Additionally, we examined new parameters, including Ghose, Veber, Egan, Muegge, and lead-likeness violations, bioavailability score, and PAINS alert. Through this analysis, we decided to retain only ligands with a PAINS alert score of 0. After removing duplicates, we obtained a refined database of 15,632 drug-like small molecules that we studied using the proposed in silico protocol (Supplementary Materials, Databases S3 and S4).

### 2.2. Ligand-Based Studies

In the initial phase of our virtual screening protocol, we used in-house-developed ligand-based techniques to efficiently analyze a large number of compounds and significantly reduce the number of candidates considered in the subsequent phases of structure-based approaches. This strategy not only enhances the reliability of the selection process but also minimizes the risk of false positives, by focusing attention on molecules with a high probability of effective interaction with the target. The use of ligand-based approaches thus represents a critical and well-reasoned step in the virtual screening workflow. It serves as an initial filter that optimizes the use of resources and research time, laying the groundwork for more in-depth structure-based studies.

To predict the binding affinity toward the selected targets, Hsp90 and ER, the Biotarget Predictor Tool (BPT), a well-established ligand-based protocol available on the DRUDIT platform (1 October 2024), was employed. Specifically, the selected targets included the Hsp90α isoform (PDB code: 2FWY), chosen for its critical role in oncogenic activity. Hsp90α is associated with adaptive responses to stress conditions that support tumor cell survival, making it a key priority for anticancer drug research [34]. The other target was the ERα isoform (PDB code: 7KBS). The decision to focus on ERα rather than ERβ stems from the predominant role of ERα in cellular proliferation processes linked to various cancers, positioning it as a crucial target for therapeutic modulation. ERα is particularly implicated in the regulation of genes associated with tumor growth and exhibits significantly higher expression in several cancers, including breast cancer. Consequently, prioritizing ERα directs the analysis toward a target of high clinical and pharmacological relevance [35,36]. Initially, ligand-based templates for Hsp90 and ER were constructed [37,38]. Extensive databases of known modulators of Hsp90 and ER were downloaded from BindingDB, which is a reliable and accessible source, containing K_i_, K_d_, IC_50_, and EC_50_ values, and target information for thousands of active molecules. In our study, we set an activity cut-off of IC_50_ < 100 nM to select the most potent inhibitors, followed by a thorough cleaning process to eliminate duplicates. These sets of inhibitors were subsequently processed using MOLDESTO (MOlecular DEScriptors Tools-1.0 version) [39], our proprietary software capable of providing over 1000 molecular descriptors (3D, 2D, and 1D) for each input structure. As a result, we obtained a “Compounds vs. Molecular Descriptors” matrix for each database, which was then converted into two sequences of descriptor pairs (mean and standard deviation) that constituted the molecular descriptor-based templates. These templates were integrated into the DRUDIT platform to evaluate the affinity of the input structures [39].

Upon completing the preliminary phase, the NCI small molecule database was subjected to BPT in Multitarget Mode (Supplementary Materials, Matrix S1). In addition to the 15,632 structures, *raloxifene* and *PF-04929113*, inhibitors of ER and Hsp90, respectively, were uploaded to DRUDIT. The molecules were analyzed using the Drudit Affinity Score (DAS), which ranges from 0 to 1. Scores close to zero indicate a low binding affinity between ligands and targets, while values close to 1 suggest a high ability of the compounds to interact with the selected targets. The DAS values of the structures for the two targets, available in Supplementary Materials, Matrix S1, were analyzed in Multitarget Mode to identify new inhibitors capable of interacting with both Hsp90/ER. The Multitarget Score (MScore) was computed through Equation (1):

(1) MScore = *DAS_ER_* × *DAS_Hsp90_*

where DAS_ER_ and DAS_Hsp90_ represent the DAS scores for the molecular descriptor-based templates of ER and Hsp90, respectively. The multitarget score allowed the selection of structures with optimal activity against both targets: the higher the two DAS scores, the higher the MScore, indicating a greater probability that the small molecule would inhibit both targets.

The compounds were ranked based on this parameter, and the MScore calculated by applying Equation (1) to the *DAS* scores of the two reference compounds *DAS_ER_* (*raloxifene*) × *DAS_Hsp90_* (*PF-04929113*) was selected as the threshold value (0.66056). The top 595 small molecules (Supplementary Materials, Matrix S2), which were predicted to have optimal interactions with both targets of interest, were then selected for further in silico structure-based studies.

### 2.3. Structure-Based Studies: Molecular Docking Analysis

In the second phase of the protocol, molecular docking studies were developed to assess the ability of the small molecules to insert into the binding sites of the target proteins. In this study, a two-step virtual docking workflow was used to further filter the compounds selected in the ligand-based analysis.

The estrogen receptor (ER) functions as a dimer and has a complex organizational structure, which is articulated into six functional domains, from the N-terminal A/B to the C-terminal F segments. The A/B sequence contains the ligand-independent transactivation domain 1 (AF-1), which, when phosphorylated, can activate the receptor independently. The C domain is highly conserved and hosts the DNA binding domain (DBD), which consists mainly of α-helices and is characterized by two zinc finger motifs. These motifs facilitate DNA binding and comprise two distinct subregions responsible for DNA recognition and receptor dimerization.

The D domain, known as the hinge region, is less conserved and serves as a link between the C and E domains. The E domain contains the ligand binding domain (LBD), which includes a ligand binding site, a co-activator/co-repressor interaction region, a dimerization interface, and the ligand-dependent transactivation domain 2 (AF-2). Finally, the F domain is a small C-terminal region that, although not essential for transactivation, appears to play a key role in protecting the receptor from proteolysis.

**Figure 1:**
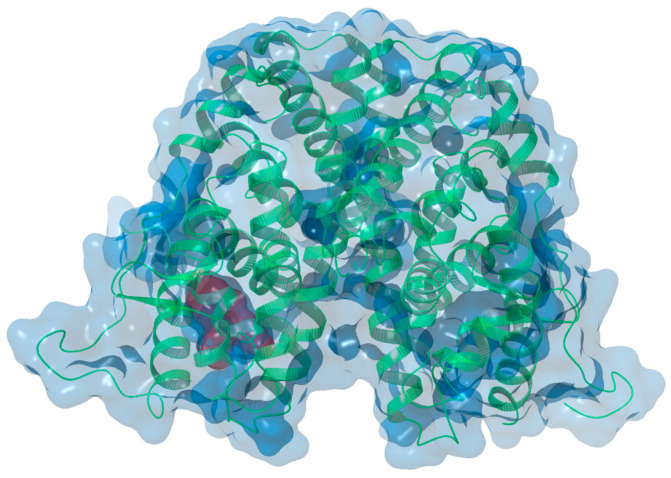
X-ray structure of the ER ligand binding pocket (PDB code 7KBS [40]).

The most important residues within the binding pocket are Ala350, Asp351, Glu353, Trp383, Leu384, Arg394, Phe404, Met421, His524, and Leu525. The hydrogen bonds established by Glu353 and Arg394 are crucial for the overall binding affinity of the ERα ligands, while the interactions provided by Asp351 are important for antagonist stabilization. Phosphorylation of other residues is of fundamental importance for various functional activities of ERα, such as hormone sensitivity, nuclear localization, DNA binding, protein/chromatin interactions, protein stability, and gene transcription. Most of these phosphorylated residues are serine residues located in the AF-1 domain (including serine residues 104, 106, 118, and 167), in the DBD (Ser236), and in the LBD (Ser305). Additionally, phosphorylation of Tyr537, located in the LBD, is also known to be important [6,7,9] (Figure 1).

**Figure 2:**
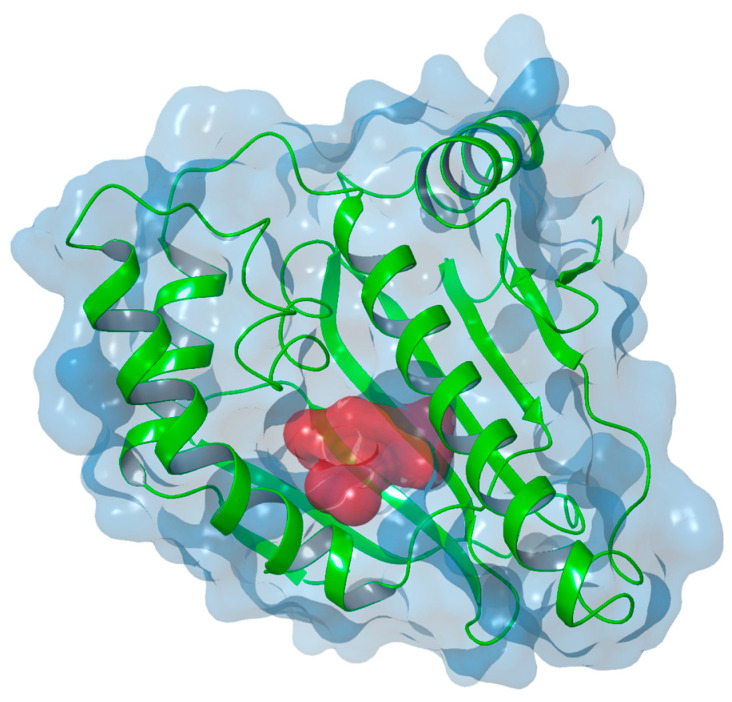
X-ray structure of the Hsp90 ligand binding pocket (PDB code 2FWY [42]).

On the other hand, Hsp90 is structurally composed of three main domains: a C-terminal domain (CTD), which contains subdomains forming Hsp90 dimers and sites for interaction with other co-chaperones; an N-terminal domain (NTD), which includes an ATP binding pocket and a subdomain that binds co-chaperones such as p50 and cdc37; a middle domain (MD) and a charged region (CR) that provide greater flexibility and dynamics to the protein [16,21,41] (Figure 2).

The docking grids were centered on the binding sites of ER and Hsp90 and included all major amino acid residues. The 595 selected small molecules were subjected to XP docking investigations (Supplementary Materials, Tables S1 and S2). For each XP docking analysis performed, the top 100 ranked small molecules were selected. Subsequently, only the structures that were common to each simulation were selected to proceed to the second step of the docking studies. In this way, 20 small molecules were identified and the docking scores for each target can be found in Table S2 of the Supplementary Materials. Surpassing the traditional rigid receptor approach in structure-based virtual screening, the Induced Fit Docking (IFD) protocol accounts for the effect of the ligand on the protein structure, providing a detailed analysis of the structural characteristics of the ligand/target complexes and their conformational changes.

In the second step of the IFD workflow, the same X-ray structures were used. The final results are reported in Tables S3 and S4 of the Supplementary Materials, while the IFD scores for the top 20 small molecules are available in Table 1.

The results of the IFD simulations indicate that several compounds can interact effectively with the target proteins, exhibiting IFD scores that are either better or comparable with the reference ligands. Among the 20 structures (Table 1), compounds **743414**, **755435**, and **676315** emerged as the best in each simulation, achieving the following IFD scores for ER: **743414** (−535.88), **755435** (−534.23), and **676315** (−533.61), all of which exceed the reference ligand *raloxifene* (−528.99).

Two reference ligands were selected for Hsp90: *PF-04929113* (IC50: 0.038 µM [43]) and the co-crystallized ligand *UP-H64* (IC50: 200 nM [42]). The choice to use two reference ligands for Hsp90 was strategic to ensure a comprehensive and robust evaluation of the interactions of the compounds with this target. *PF-04929113* was selected for its high biological activity, indicating strong affinity and potency, making it an ideal benchmark for comparing the efficacy of new compounds. Conversely, the co-crystallized ligand *UP-H64* was chosen due to the availability of its crystallographic structure complexed with Hsp90 in the Protein Data Bank (PDB), allowing for more accurate modeling of protein–ligand interactions during the docking simulations and providing a well-defined structural context for interpretation the IFD scores.

Compounds **676315** (−479.944) and **755435** (−478.905) demonstrated better IFD scores than the two reference ligands, *PF-04929113* (−478.025) and the co-crystallized ligand *UP-H64* (−474.866). However, compound **743414** (−476.988) exhibited a lower IFD score than *PF-04929113* but a higher score than the co-crystallized ligand *UP-H64*. Considering the data obtained from the IFD simulations, compounds **743414**, **755435**, and **676315** emerged as potential multitarget candidates, which is why they were subjected to further molecular dynamics analyses.

### 2.4. Molecular Dynamics Simulations

Molecular dynamics simulations were performed for the three best compounds to gain insights into the structural characteristics of compounds **743414**, **676315**, and **755435** in complex with the two targets, ER (PDB code 7KBS) and Hsp90 (PDB code 2FWY), using *raloxifene* and *UP-H64* as reference compounds. The Root Mean Square Deviation (RMSD) was calculated for both the ligands and the proteins during a 100 ns simulation trajectory for each ligand/protein complex. This parameter was used to assess the stability and convergence of the simulations by measuring the average change in the displacement of the protein backbone from the initial frame (t = 0) compared to a reference frame.

**Figure 3:**
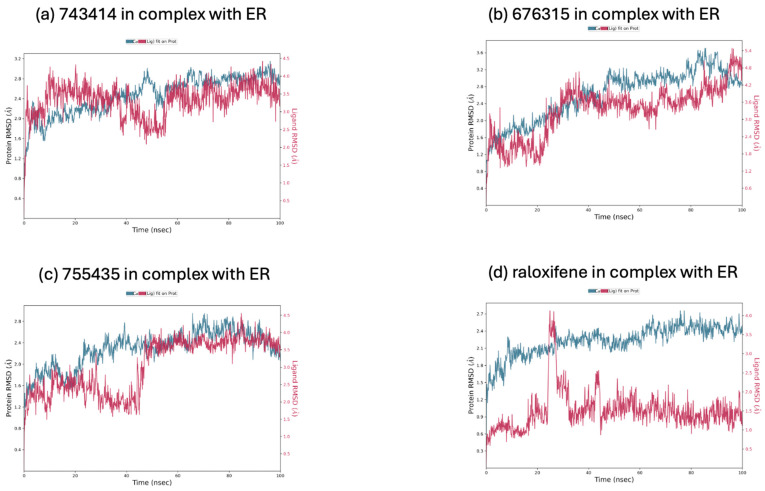
(**a**) Calculated RMSD during 100 ns of the simulation trajectory for the **743414**/ER complex; (**b**) calculated RMSD during 100 ns of the simulation trajectory for the **676315**/ER complex; (**c**) calculated RMSD during 100 ns of the simulation trajectory for the **755435**/ER complex; (**d**) calculated RMSD during 100 ns of the simulation trajectory for the *raloxifene*/ER complex.

In Figure 3a–d, the RMSD plots for compounds **743414**, **676315**, and **755435** and *raloxifene* in complex with the ER target are shown. The left *Y*-axis indicates the RMSD evolution of the ER. The analysis shows that the RMSD of the protein varies within the acceptable range of 1–3 Å, suggesting acceptable structural stability. The right *Y*-axis, on the other hand, indicates the RMSD of the various analyzed ligands. The RMSD values of the ligands are not significantly higher than the RMSD values of the ER, suggesting that the ligands did not diffuse from their initial binding site during the simulation. An exception is graph b, where the ligand **676315** exhibits RMSD values between 0 and 4 Å, as well as excessive fluctuations during the simulation time.

Furthermore, convergence between the RMSD of the protein and the ligand, a crucial parameter indicating the equilibrium of the system, was achieved in the complexes **743414**/ER and **755435**/ER at the end of the simulations. This indicates that these systems reached a stable configuration. On the other hand, in the *raloxifene*/ER complex, there was no clear convergence between the RMSD values of the protein and the reference ligand, suggesting that equilibrium might not have been fully achieved and indicating a potentially lower stability in the timescale considered.

**Figure 4:**
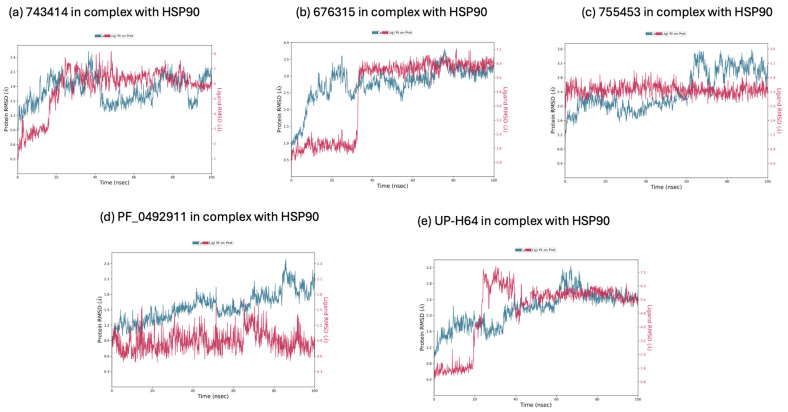
(**a**) Calculated RMSD during 100 ns of the simulation trajectory for the **743414**/Hsp90 complex; (**b**) calculated RMSD during 100 ns of the simulation trajectory for the **676315**/Hsp90 complex; (**c**) calculated RMSD during 100 ns of the simulation trajectory for the **755435**/Hsp90 complex; (**d**) calculated RMSD during 100 ns of the simulation trajectory for the *PF-04929113*/Hsp90 complex; (**e**) calculated RMSD during 100 ns of the simulation trajectory for the *UP-H64*/Hsp90 complex.

The RMSD was also calculated for the 100 ns simulation trajectory of the same ligands and the Hsp90 protein, with the aim of measuring the average change in backbone displacement. Analyzing the graphs shown in Figure 4a–e, we observe on the left *Y*-axis the evolution of the RMSD of the Hsp90 protein, which varies between 1 and 3.5 Å. The right *Y*-axis shows the RMSD values of the ligand; specifically, the ligands **743414**, **676315**, and *UP-H64* exhibit high RMSD values (0–5 Å) compared to the RMSD values of the protein. The ligand *PF-04929113* (graph d) shows RMSD values within the acceptable range of 0–2.4 Å. Nonetheless, convergence between the RMSD values of the protein and *PF-04929113* is not achieved. In graph c, both the protein and the ligand **755435** have reached acceptable RMSD values, indicating good convergence between the two, which translates into perfect stability of the **755435**/Hsp90 complex. These analyses suggest that the compound **755435** achieves good stability in both binding sites of the protein, thereby confirming the potential to inhibit both molecular targets. For this reason, we conducted further analyses on the compound **755435** in complex with ER and Hsp90, respectively.

**Figure 5:**
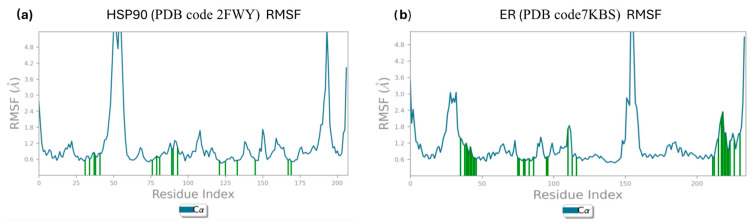
(**a**) Calculated P-RMSF during the simulation for Hsp90 in complex with **755435**; (**b**) calculated P-RMSF during the simulation for ER in complex with **755435**.

The Protein Root Mean Square Fluctuation (P-RMSF), a valuable tool for characterizing local changes along the protein chain, was calculated for each specific residue. Figure 5a,b present the P-RMSF plots for each protein (Hsp90 and ER in complex with **755435**, respectively), where the peaks represent regions of the protein that fluctuate the most during the simulation. Notably, fluctuations at the N and C termini compared to secondary structural elements, such as alpha helices and beta sheets, which usually exhibit greater rigidity, are of particular interest. In plot a, related to Hsp90, significant peaks are observed around residues 50 and 190, with fluctuations exceeding 4.8 Å. These peaks suggest high-mobility regions, likely corresponding to loops or N and C termini, which tend to be more flexible. In plot b, related to ER, significant peaks are also found around residues 50 and 200, with fluctuations up to 4.8 Å.

Protein residues interacting with the ligand are marked by green vertical bars, facilitating the interpretation of molecular dynamics data. It is noteworthy that many of these bars are in regions with low fluctuations (RMSF < 1.2 Å). This is consistent with the fact that residues forming the binding site tend to be more rigid, contributing to the stability of the ligand–protein interaction. In the Supplementary Materials (Figures S6–S8), the Protein Root Mean Square Fluctuation (P-RMSF) profiles of the reference ligands—*raloxifene* in complex with ER, and *PF-04929113* and *UP-H64* in complex with Hsp90—are provided. The secondary structural elements of Hsp90 and ER, such as alpha helices and beta sheets, are observed throughout the simulation. The results related to the composition of secondary structural elements for each frame of the trajectory are reported in Figures S1 and S2 of the Supplementary Materials. This analysis provides a detailed understanding of the protein regions that are most mobile during the simulation, highlighting the importance of ligand-interacting residues in maintaining the stability of the protein complex.

**Figure 6:**
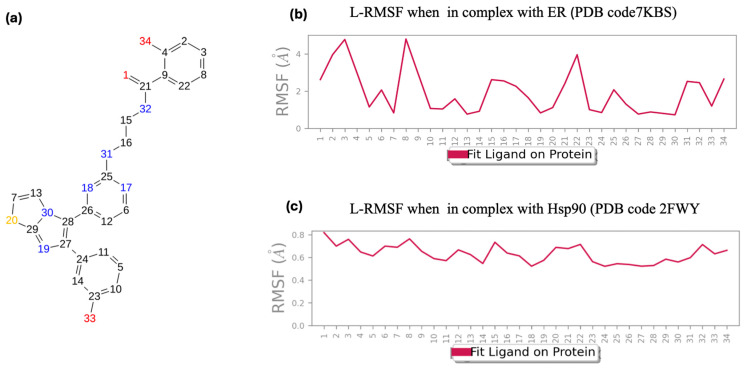
(**a**) Two-dimensional structure of compound **755435**; (**b**) calculated L-RMSF during the simulation for **755435** in complex with ER; (**c**) calculated L-RMSF during the simulation for **755435** in complex with Hsp90.

The Ligand Root Mean Square Fluctuation (L-RMSF) is a critical metric for assessing the mobility of ligand atoms, allowing the identification of regions exhibiting significant variations over time. This analysis is crucial for understanding the conformational entropy of the ligand and its contribution to the binding process. For compound **755435**, the L-RMSF was calculated within each complex formed with ER and Hsp90, setting the reference time (tref) to the first frame as the zero-time point. The L-RMSF results, presented in Figure 6b,c for ER and Hsp90, respectively, provide a detailed view of the ligand’s atomic fluctuations, with a 2D visual representation shown in Figure 6a.

This approach isolates the ligand’s intrinsic variations, eliminating the effects of the global fluctuations of the complex. Compound **755435**, within the protein environment, demonstrates significant stability, with RMSF values within an acceptable range.

Specifically, in graph b, related to the ER complex, significant variations in atomic fluctuations are observed along the entire structure of the ligand. Some atoms exhibit high fluctuations, indicating greater mobility in those specific regions. The highest L-RMSF peaks suggest that certain functional groups of the ligand have high conformational entropy, implying possible dynamic interactions with the binding pocket residues of ER. In graph C, related to the Hsp90 complex, the atomic fluctuations of the ligand are relatively lower than those observed in the ER complex. This suggests that ligand **755435** maintains greater stability within the binding pocket of Hsp90. The lower overall L-RMSF indicates strong and specific interactions between the ligand and the residues of the binding pocket, contributing to the stability of the complex.

In general, low L-RMSF values indicate high ligand stability in the binding pocket, suggesting strong and specific interactions with the protein. In summary, the L-RMSF analysis provides valuable insights into the conformational stability of the ligand and its interaction with the target protein, highlighting the potential of compound **755435** as a stable candidate for further structural and functional studies. In the Supplementary Materials (Figures S9–S11), the Ligand Root Mean Square Fluctuation (L-RMSF) profiles were calculated for *raloxifene* in complex with ER, as well as for *PF-04929113* and *UP-H64* in complex with Hsp90.

Additionally, during the simulation, the ligand torsional profiles, summarizing the conformational evolution of each rotatable bond in ligand **755435**, was carefully analyzed, and the results are reported in Figure S3 of the Supplementary Materials.

Subsequently, several structural parameters were calculated for each complex to provide a comprehensive analysis of their molecular characteristics. Specifically, the Radius of Gyration (rGyr) was calculated to measure the ligand’s extent, equivalent to its principal moment of inertia. Additionally, the Intramolecular Hydrogen Bonds (intraHBs), Molecular Surface Area (MolSA), Solvent Accessible Surface Area (SASA), and Polar Surface Area (PSA) were determined. The results of these calculations are presented in Figures S4 and S5 of the Supplementary Materials.

### 2.5. Integrated Analysis of Amino Acid Interactions Between Compound **755435** and Protein Binding Sites

In light of the results obtained from molecular dynamics simulations, we conducted a detailed analysis of the key interactions between the best docked pose of compound **755435** and the amino acid residues for each protein binding site (see Table 2 and related 3D binding site figures). This in-depth analysis of amino acid interactions, performed through the study of the best docked pose derived from IFD analysis and molecular dynamics studies, was crucial for understanding the potential mechanism of action of the compound with respect to ER and Hsp90. Integrating the results of IFD and molecular dynamics simulations offers a dual advantage: on the one hand, IFD provides a detailed snapshot of the static interactions in the optimal binding configuration, while molecular dynamics simulations allow for the observation of the stability and flexibility of these interactions over time. This combined approach is particularly useful for identifying the key contributions of amino acid residues to the stability of the molecule–target complex, thereby enhancing our understanding of the molecular dynamics and potential therapeutic applications of the compound.

**Figure 7:**
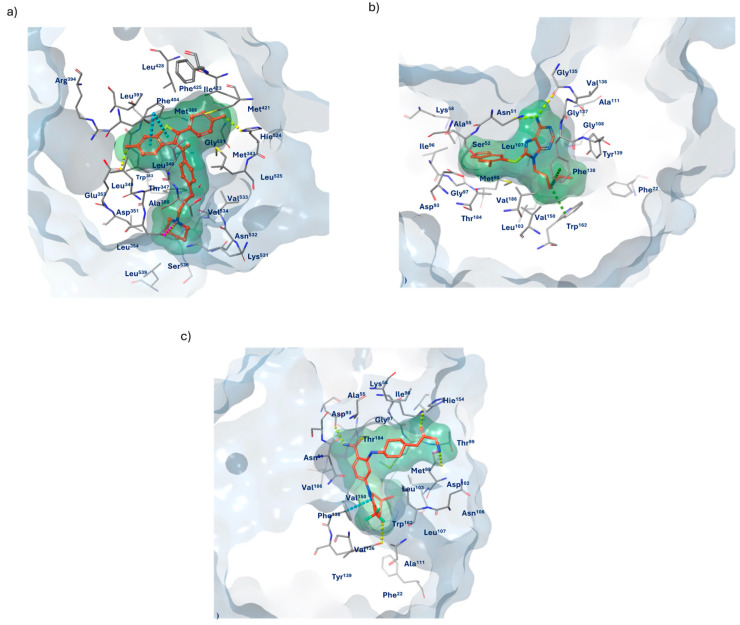
(**a**) Three-dimensional binding site of ER in complex with *raloxifene* (PDB code 7KBS); (**b**) Hsp90 in complex with *UP-H64* (PDB code 2FWY); (**c**) Hsp90 in complex with *PF-04929113* (PDB code 2FWY). Interaction color legend: yellow—hydrogen bonds; pink—salt bridge; green—Pi–cation interaction; blue—Pi-Pi stacking interaction.

Figure 7a shows ER binding site (PDB code 7KBS) in complex with *raloxifene*. *Raloxifene* is a selective estrogen receptor modulator (SERM) capable of acting both as an agonist and an antagonist of the ER in various tissues. The drug interacts with specific amino acid residues located in the ligand binding domain (LBD) of the estrogen receptor. Notable residues include Ala^350^, Asp^351^, Glu^353^, Trp^383^, Leu^384^, Arg^394^, Phe^404^, Met^421^, His^524^, and Leu^525^ [6]. Two key residues, Glu^353^ and His^524^, located at opposite ends of the binding pocket of the receptor, are crucial for ligand recognition and hydrogen bond formation. The amide group of Glu^353^ forms a hydrogen bond with the hydroxyl group on the benzothiophene ring of *raloxifene* (O-H---N), a critical interaction for the stability of the complex and for inducing the receptor conformation necessary for the drug to exert its effects as a selective modulator. His^524^, through its imidazole group, forms a hydrogen bond with the hydroxyl group of the phenolic ring (O-H---N). Additionally, the carboxyl group of the Asp^351^ side chain forms an H-bond with the nitrogen of the piperidine ring (N-H---O), which is essential for ligand stabilization. Beyond the hydrophilic residues at the ends of the binding pocket, the rest of the pocket is highly hydrophobic. Notably, Phe^404^ engages in π-π interactions with the benzothiophene ring, significantly contributing to the ligand–receptor complex stability. Figure 7b depicts Hsp90 in complex with the co-crystallized ligand *UP-H64* (PDB code 2FWY). Hsp90 is a homodimeric protein characterized by three main domains, with the N-terminal domain (NTD) playing a crucial role. This domain includes the ATP binding site, which is essential for protein function. Within this site, several amino acid residues are critical for ligand interactions, such as Trp^162^ and Phe^138^. These residues participate in hydrophobic π-cationic interactions with the amine group of the co-crystallized ligand side chain, contributing to the complex’s stability. The carbonyl oxygens of Gly^135^ and Asn^51^ are involved in hydrogen bond formation with the amine group of adenine, facilitating ATP binding (N-H---O; N-H---O, respectively). Val^136^, Tyr^139^, Phe^22^, Leu^103^, Val^150^, Ile^96^, Val^186^, and Met^98^ constitute the protein’s hydrophobic pocket, contributing to its structural stability and ligand recognition. The second reference ligand, *PF-04929113*, in complex with Hsp90 (Figure 7c), forms a number of interactions comparable to those of the co-crystallized ligand *UP-H64.* Indeed, the *PF-04929113*/Hsp90 complex also exhibits significant π-π stacking interactions between Phe^138^ and the indazole of the ligand, which contributes to the stabilization of the ligand–protein complex. Various carbonyl groups in the molecule form hydrogen bonds with the amine group of the Lys^58^ side chain (N-H---O) and with the hydroxyl group of the Thr^184^ side chain (O-H---O). Additionally, the phenolic group of the Tyr^139^ side chain forms an H-bond with the carbonyl group of the ligand (O-H---O). Additionally, other hydrogen bonds can be observed between the oxygen of Asp^93^ and the amide portion of the compound (O---H-N), and between Asp^102^ and the amine group.

**Figure 8:**
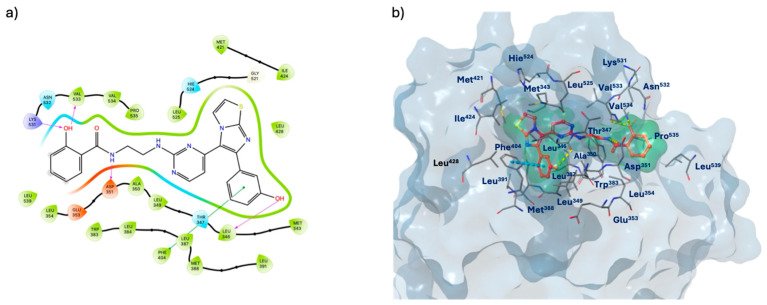
(**a**) Two-dimensional structure of the detailed interactions between the atoms of ligand **755435** and the protein residues of ER; (**b**) three-dimensional binding site of **755435** in complex with ER (PDB code 7KBS).

The selected derivative, **755435**, although forming a total number of interactions lower than the reference ligands such as *raloxifene*, *PF-04929113,* and *UP-H64* (Table 2), still meets the essential binding requirements with ER and Hsp90 receptors, at approximately 4 Å. In the ligand binding domain (LBD) of the ER (Figure 8), compound **755435** establishes significant interactions with all the crucial residues of the active site. A key element shared between the binding mechanisms of *raloxifene* and compound **755435** is the formation of hydrogen bonds at opposite ends of the ER binding pocket, which contributes to the stabilization of the ligand–receptor complex. The oxygen of the carbonyl group of Val^533^ and the amine group of Lys^531^ form a hydrogen bond with the phenolic group of **755435** (O---H-O; N-H---O, respectively). On the opposite side of the receptor’s binding site, the oxygen of the carbonyl group from the Leu^346^ backbone forms a hydrogen bond with the phenolic group of **755435**, where the oxygen of the carbonyl group acts as an acceptor and the phenolic group of the compound acts as a donor (O-H---O). Additionally, the phenolic ring is further stabilized through a π-π stacking interaction with residue Phe^404^. Another hydrogen bond is observed between the amide portion of **755435** and the carboxyl group of Asp^351^ (N-H---O). Finally, compound **755435** is surrounded by a network of amino acids in the binding site, including Leu^539^, Leu^354^, Trp^383^, Leu^384^, Ala^350^, Leu^349^, Leu^387^, Met^388^, Leu^391^, Met^343^, Leu^428^, Ile^424^, Met^421^, Leu^525^, Pro^535^, and Val^354^. This network contributes to creating a hydrophobic environment that further supports the interaction and stabilization of the compound within the binding site of the receptor. 

**Figure 9:**
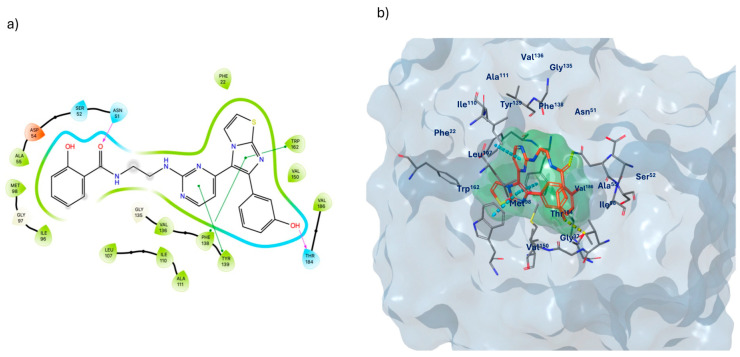
(**a**) Two-dimensional structure of the detailed interactions between the atoms of ligand **755435** and the protein residues of Hsp90; (**b**) three-dimensional binding site of **755435** in complex with Hsp90 (PDB code 2FWY).

For Hsp90 as well (Figure 9), compound **755435** meets the essential requirements for effective binding to the protein compared to the reference ligands, *PF-04929113* and *UP-H64*. Specifically, compound **755435** is involved in the formation of two hydrogen bonds: one between the amine group of Asn^51^ and the carbonyl group of compound **755435** (N-H---O), and the second between the oxygen of the Thr^184^ backbone and the phenolic group of the compound (O-H---O). Additionally, Trp^162^ and Phe^138^ are involved in π-π stacking interactions, with the pyrimidine ring of the compound aligning with residue Tyr^139^, and the imidazole portion of the imidazothiazole system being stabilized by interactions with Phe^138^ and Trp^162^. Furthermore, the amino acids Phe^22^, Val^150^, Val^186^, Val^136^, Ala^111^, Ile^110^, Leu^107^, Ile^96^, Met^98^, and Ala^55^ form the hydrophobic network surrounding the binding site.

**Figure 10:**
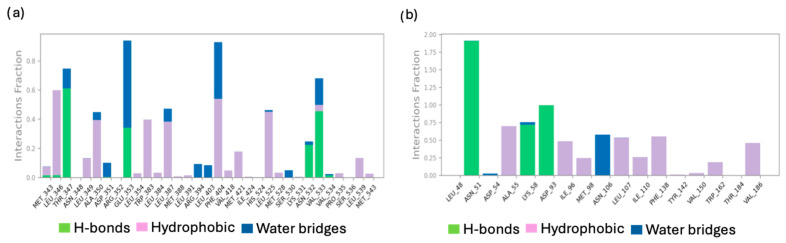
(**a**) Protein–ligand interactions examination across the simulation time for **755435**/ER complex; (**b**) protein–ligand interactions examination across the simulation time for **755435**/Hsp90 complex.

Molecular dynamics simulations also present graphs of protein–ligand contacts and explain the interaction fraction of protein residues with the ligand, indicating the percentage of simulation time that the specific interaction between the ligand and the receptor complexes is maintained. Figure 10a,b show the protein–ligand contacts for the **755435**/ER and **755435**/Hsp90 complexes, respectively.

The **755435**/ER complex (Figure 10a) maintained strong interactions with Leu^346^, Thr^347^, Ala^350^, Glu^353^, Phe^404^, Leu^525^, and Val^533^. Conversely, for the **755435**/Hsp90 complex (Figure 10b), Ala^55^, Lys^58^, Asp^93^, and Phe^138^ exhibited the highest interaction fractions, with Asn^51^ varying between 1.75 and 2.

It is noteworthy that compound **755435**, identified through our hybrid ligand structure-based protocol as a potential dual-target inhibitor against Hsp90 and ER, has demonstrated biological activity against several breast cancer cell lines including ER+ breast cancer, such as MCF7 and T-47D [44]. This underscores the effectiveness of integrating our ligand-based tools with conventional structure-based techniques, establishing it as a reliable and robust approach for screening large compound libraries in the field of targeted drug discovery.

## 3. Materials and Methods

### 3.1. Database Cleaning Phase

In silico prediction of pharmacokinetic properties is a pivotal component of drug discovery. This study employed two prominent computational tools, QikProp and SwissADME, to evaluate the pharmacokinetic profiles of the designed compounds and to eliminate those lacking drug-likeness properties. QikProp, a module within the Schrödinger Suite, leverages molecular descriptors to predict various drug-like properties, such as solubility, permeability, and bioavailability. The calculations were executed using default settings, and the resulting output provided valuable insights into the ADME (Absorption, Distribution, Metabolism, and Excretion) characteristics of the compounds. Furthermore, SwissADME, an online platform developed by the Swiss Institute of Bioinformatics, was utilized to assess additional pharmacokinetic parameters. SwissADME employs a robust set of algorithms to estimate physicochemical properties, drug-likeness, and medicinal chemistry-related parameters. The combined analyses from QikProp and SwissADME offered a thorough understanding of the potential drug-like properties of the investigated compounds, facilitating the identification of lead candidates for subsequent experimental validation.

### 3.2. Ligand-Based Protocols

The web service DRUDIT (www.drudit.com, accessed on 1 October 2024) operates on four servers, each capable of handling over ten tasks simultaneously, utilizing various software modules written in C and JAVA on MacOS Mojave. The Biotarget Finder Module was employed in Multitarget Mode to screen the extensive, curated NCI database of active small molecules as potential ER and HSP90 inhibitors for breast cancer treatment [39].

The Biotarget Predictor Tool (BPT) predicts the binding affinity between candidate molecules and specific biological targets. Templates for ER and HSP90 were constructed using sets of well-known protein inhibitors with affinities below 100 nM, sourced from BindingDB [45]. Molecular descriptors were calculated using MOLDESTO. The five developed molecular descriptor target templates were integrated into DRUDIT, and the default DRUDIT parameters (N = 500, Z = 50, G = a) were applied [39,45,46]. During the initial phase of the in silico workflow, the cleaned NCI database was uploaded to DRUDIT and submitted to the Biotarget Predictor in Multitarget Mode. The output results were presented as DAS (Drudit Affinity Score) values for each structure, indicating the binding affinity of compounds against both ER (DASER) and HSP90 (DASHSP90).

### 3.3. Structure-Based Studies

The preparation of ligands and proteins for in silico studies followed the detailed procedures outlined below.

#### 3.3.1. Ligand Preparation

The ligands intended for docking studies were carefully processed utilizing the LigPrep tool from Schrödinger’s Maestro Suite (Schrödinger Release 2017-2, LigPrep; Schrödinger, LLC: New York, NY, USA, 2017). For each ligand, all possible stereoisomers and tautomers were generated under physiological conditions (pH 7.0 ± 0.4), following the default parameters and using the Epik method for ionization [41]. Afterward, the ligands underwent energy minimization, with the OPLS 2005 force field applied to ensure optimal stability [42].

To prepare the ligands for docking simulations, they were subjected to the LigPrep protocol within the Schrödinger Maestro platform. This involved generating every likely tautomer and stereoisomer at a pH of 7.0 ± 0.4, following standard settings and the Epik ionization technique. Subsequently, the ligands were energy-minimized using the OPLS 2005 force field to obtain their lowest energy conformation.

#### 3.3.2. Protein Preparation

Crystal structures of ER and HSP90 (PDB code7KBS and PDB code 2FWY, respectively) were sourced from the Protein Data Bank [47,48] and underwent preparation using the Protein Preparation Wizard in the Schrödinger software (Schrödinger Suite 2017-2 Protein Preparation Wizard; Epik, Schrödinger, LLC: New York, NY, USA, 2017), adhering to default settings [49]. This involved assigning bond orders, including the Het group, deleting all water molecules, and protonating heteroatom states using the Epik tool, with the pH set at biologically relevant values (7.0 ± 0.4). Optimization of the H-bond network ensued, and the structures underwent a restrained energy minimization step (RMSD of the atom displacement for terminating minimization was set at 0.3 Å), employing the OPLS 2005 force field [50].

#### 3.3.3. Docking Validation

Molecular docking studies were performed and scored using the Glide module within the Schrödinger Suite (Schrödinger Release 2024-4: Glide, Schrödinger, LLC, New York, NY, USA, 2024). Receptor grids were generated by designating the original ligands—raloxifene (for ER, PDB code 7KBS) and UP-H64-LIGCOCRYST (for HSP90, PDB code 2FWY)—as the centroids of the grid boxes. Utilizing the Extra Precision (XP) mode for scoring, 3D conformers were docked into the receptor models. A post-docking minimization step was applied to each ligand conformer, producing a maximum of two docking poses and up to five poses per ligand conformer. Remarkably, the docking protocol successfully redocked the original ligands within the receptor binding pockets with an RMSD < 0.51 Å.

The Extra Precision (XP) Docking was employed to preliminarily screen compounds selected by DRUDIT. Subsequently, the Induced Fit Docking (IFD) simulation was conducted using the Schrödinger IFD application, a precise and robust method accommodating the flexibility of both ligand and receptor [51,52]. Applying Schrödinger’s validated IFD protocol, the ER and HSP90 proteins (PDB codes7KBS, and 2FWY, respectively), previously refined by the Protein Preparation module, were used. The IFD score, calculated as IFD score = 1.0 Glide Gscore + 0.05 Prime Energy, incorporating protein–ligand interaction energy and total system energy, was used to rank the IFD poses.

#### 3.3.4. Molecular Dynamics Simulations: Details of the Experimental Procedure

Molecular dynamics simulations were conducted to investigate the effects of the solvent system on the stability of the protein–ligand complex structure. These simulations were carried out using the explicit solvent molecular dynamics package Desmond, provided by Schrödinger in Maestro version 13.8.155, MMshare version 6.4.195, Release 2023–4, available for the Linux-x8564 platform. The simulations were executed on a Dell Inc. (Round Rock, TX, USA) Precision 7960 Tower equipped with an Intel^®^ Xeon^®^ w9-3475X processor (72 cores) and an NVIDIA Corporation graphics processing unit running on Ubuntu 22.04.4 LTS 64-bit.

The molecular dynamics simulations were performed using the top docking poses of each ligand (**755435**, **743474**, and **676315**) in complex with the target receptors of interest (Hsp90 and ER), retrieved from Induced Fit Docking studies, to confirm the stability and binding strength of the ligand/target complexes. Simulations were conducted for a simulation time of 100 ns, generating approximately 1000 frames in the trajectory.

These simulations were performed under the constant temperature and pressure (NPT) ensemble, allowing precise control of temperature and pressure. In the NPT ensemble, pressure adjustments were made by altering the volume, and the unit cell vectors were allowed to change freely. The simulation parameters were set with a system temperature of 300 K and a pressure of 1.01325 bar. Subsequently, 100 ns production runs were performed for the various complexes. At the conclusion of each simulation, the output file was analyzed using the Simulation Interaction Diagram tool. This tool provides graphical representations of all calculated parameters during the simulations, including Root Mean Square Deviation (RMSD) for the protein (Cα, backbone, side chains, or heavy atoms) and ligands; Root Mean Square Fluctuation (RMSF) for protein residues and ligands; protein–ligand contacts; and ligand torsion. The graphical depictions of the simulations reported in both the manuscript and the supporting information were generated using this tool.

## 4. Conclusions

Dual inhibition of estrogen receptors (ERs) and the chaperone protein Hsp90 emerges as a promising therapeutic strategy for the treatment of ER+ breast cancer, particularly in cases of resistance to conventional endocrine therapies. In our study, we integrated both ligand-based and structure-based virtual screening approaches to facilitate the identification of small molecules with high dual inhibition potential.

Initially, we refined the NCI database using tools such as QikProp and SwissADME to ensure data quality. The refined database was then subjected to ligand-based studies using the Biotarget Predictor Tool (BPT) in Multitarget Mode. This phase enabled us to select compounds with potential for interaction with both targets.

Molecular dynamics simulations further confirmed that the selected compounds, particularly compound **755435**, exhibit favorable interactions with the binding sites of ER and Hsp90. Compound **755435** stands out for its ability to simultaneously interact with both targets, suggesting a potential mechanism of action based on multitarget inhibition.

The significance of developing multitarget drugs lies in their capacity to address multiple concurrent pathological pathways, which is especially crucial in cases of therapeutic resistance. By simultaneously inhibiting ER and Hsp90, a multitarget drug can not only tackle the primary tumor growth mechanism mediated by ER but also interfere with cellular stress mechanisms and the stabilization of oncoproteins mediated by Hsp90. This approach may reduce the risk of developing resistance, enhance therapeutic efficacy, and potentially decrease the need for complex and costly drug combinations.

## Figures and Tables

**Table 1:** IFD scores of the top 20 selected small molecules and the reference co-crystallized ligand *raloxifene* against ER (PDB code 7KBS) and the reference ligands *PF-04929113*, *UP-H64* against HSP90 (PDB code 2FWY).

ER		HSP90	
Title	IFD Score	Title	IFD Score
**743414**	−535.88	**676315**	−479.944
**755435**	−534.23	**755435**	−478.905
**676315**	−533.61	*PF-04929113*	−478.025
*raloxifene*	−528.99	**743414**	−476.988
**623292**	−526.93	**732491**	−475.472
**732491**	−526.74	**678359**	−475.108
**700643**	−526.08	**623292**	−475.065
**667933**	−525.91	*UP-H64*	−474.866
**695838**	−525.91	**695838**	−474.864
**694110**	−525.65	**667933**	−473.692
**679211**	−525.45	**700643**	−472.682
**670869**	−525.38	**694110**	−472.341
**678359**	−524.81	**679211**	−471.944
**679210**	−524.74	**670869**	−471.328
**679208**	−524.61	**679210**	−471.286
**736041**	−524.29	**665690**	−470.545
**665690**	−523.8	**679208**	−470.348
**729154**	−522.93	**729154**	−470.303
**635321**	−521.56	**736041**	−469.883
**635313**	−519.18	**635313**	−469.768
		**635321**	−469.067

**Table 2:** Overview of the amino acids involved in the binding of the selected compound 755435 in the binding sites of ER and Hsp90, compared to ligands raloxifene, *PF-04929113* and *UP-H64*, at 4 Å proximity.

ER			Hsp90			
Title	*raloxifene*	755435	Title	*PF-04929113*	*UP-H64*	755435
Met^343^		X	Phe^22^	X	X	X
Leu^346^	X	*X	Asn^51^	X	*X	*X
Thr^347^	X	X	Ser^52^	X	X	X
Leu^349^	X	X	Asp^54^			X
Ala^350^	X	X	Ala^55^	X	X	X
Asp^351^	*X	*X	Lys^58^	*X	X	
Glu^353^	*X	X	Asp^93^	*X	X	
Leu^354^	X	X	Ile^96^	X	X	X
Trp^383^	X	X	Gly^97^	X	X	X
Leu^384^	X	X	Met^98^	X	X	X
Leu^387^	X	X	Thr^99^	X		
Met^388^	X	X	Asp^102^	*X	X	
Leu^391^	X	X	Leu^103^	X	X	
Arg^394^	X		Asn^106^	X		
Phe^404^	#X	#X	Leu^107^	X	X	X
Met^421^	X	X	Gly^108^		x	
Ile^424^	X	X	Ile^110^			X
Phe^425^	X		Ala^111^	X	X	X
Leu^428^	X	X	Gly^135^		*X	X
Gly^521^	X	X	Val^136^	X	X	X
Hie^524^	*X	X	Gly^137^		X	
Leu^525^	X	X	Phe^138^	#X	#X	#X
Lys^531^	X	*X	Tyr^139^	*X	X	#X
Asn^532^	X	X	Val^150^	X	X	X
Val^533^	X	*X	Hie^154^	X		
Val^534^	X	X	Trp^162^	X	#X	#X
Pro^535^	X	X	Thr^184^	*X	X	*X
Ser^536^	X		Val^186^	X	X	X
Leu^539^	X	X				
Tot.	28	27		23	23	19

*X: H-bonds; #X: hydrophobic interaction.

## Data Availability

Data are contained within the article and Supplementary Materials.

## Supplementary Materials

The following supporting information can be downloaded at: https://www.mdpi.com/article/10.3390/molecules29246040/s1, Databases S1 and S2: NCI database; Databases S3 and S4: Refined NCI database; Matrix S1 and S2: DAS; Tables S1 and S2: Docking score; Tables S3 and S4: IFD score; Figures S1–S11: Molecular dynamics simulations.
