# Supplementary material for: In Silico Design of Dual Estrogen Receptor and Hsp90 Inhibitors for ER-Positive Breast Cancer Through a Mixed Ligand/Structure-Based Approach
Source: Molecules. 2024 Dec 21;29(24):6040. doi: 10.3390/molecules29246040 (PMC11676166; doi:10.3390/molecules29246040)
Supplement: Supplementary file 1 [file molecules-29-06040-s001.zip › Supplementary Material.pdf]

Figures S1-S2 provide a summary of the tracked protein Secondary Structure Elements (SSE) for HSP90 and ER, respectively. The Figures S1a and S2a plots illustrate the distribution of SSE by residue index across the entirety of the protein structure for Hsp90 and ER, respectively. Directly below, the subsequent Figures S1b and S2b plots offer a comprehensive summary of the SSE composition for each trajectory frame, providing a dynamic snapshot of structural changes over the simulation duration. Finally, the lowermost plot in Figures S1c and S2c monitor the SSE assignment for each residue over time, offering a detailed, time-resolved perspective on the evolution of secondary structure elements. Together, these plots provide a thorough and multi-faceted analysis of SSE dynamics, contributing to a comprehensive understanding of the protein structural behavior throughout the molecular dynamic simulations.

a)

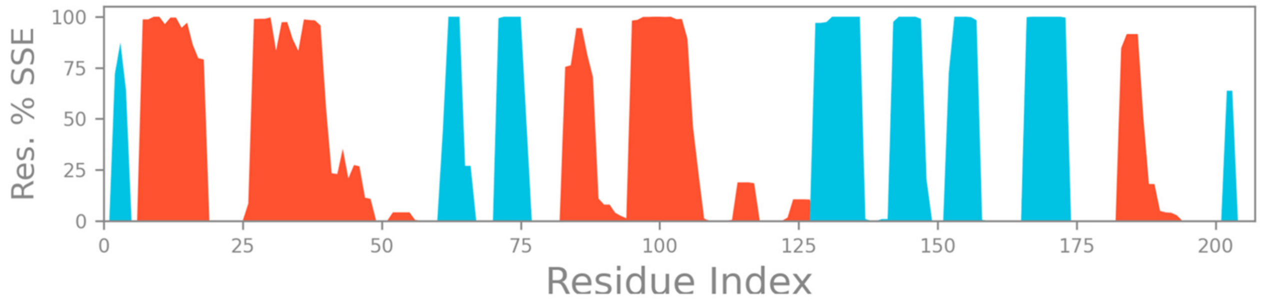

b)

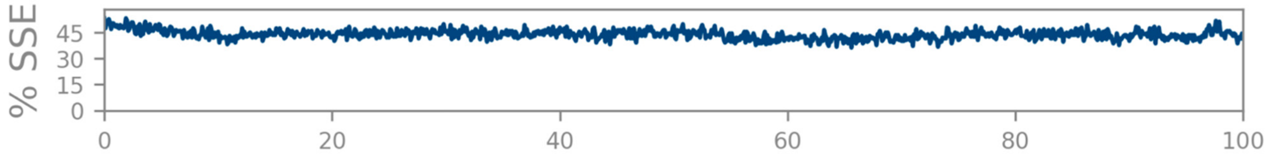

c)

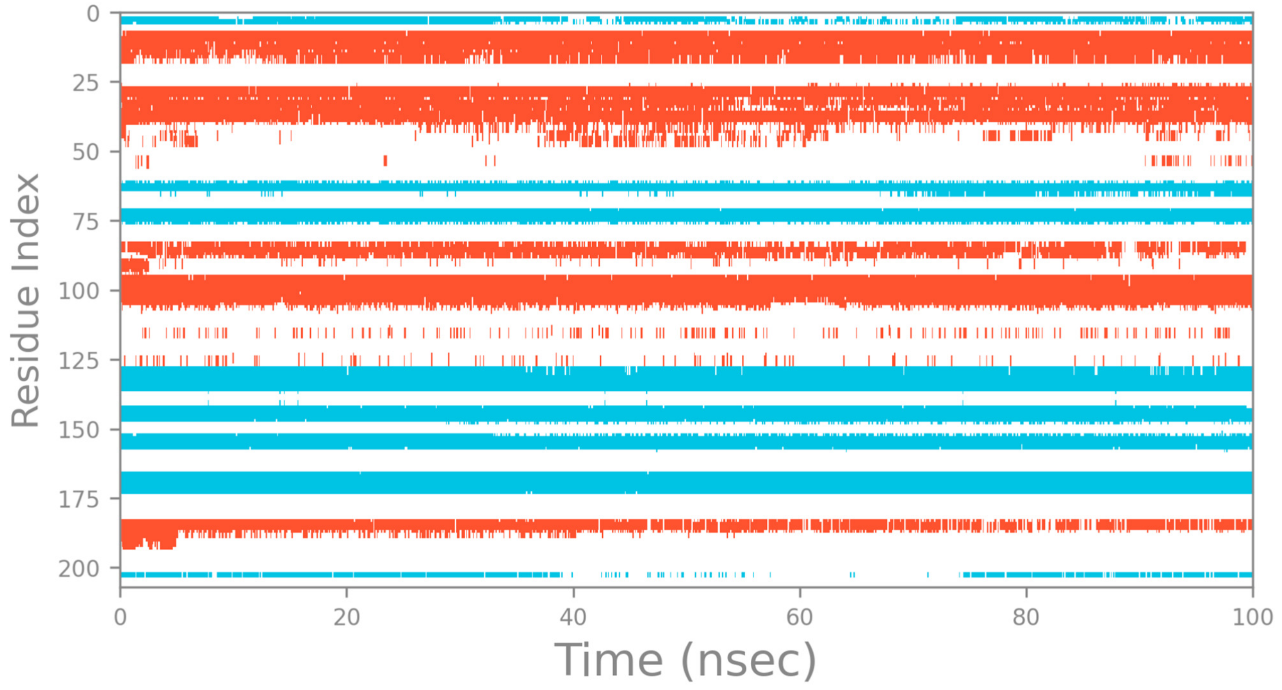

Figure S1. Hsp90 protein secondary structure elements (SSE) evaluation: **(a)** distribution of SSE by residue index across the entirety of the protein structure; **(b)** dynamic snapshot of structural changes over the simulation duration; **(c)** SSE assignment for each residue over time.

a)

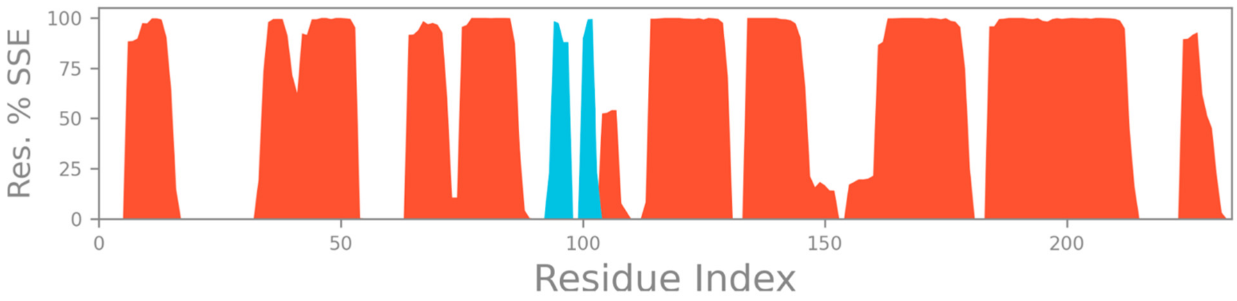

b)

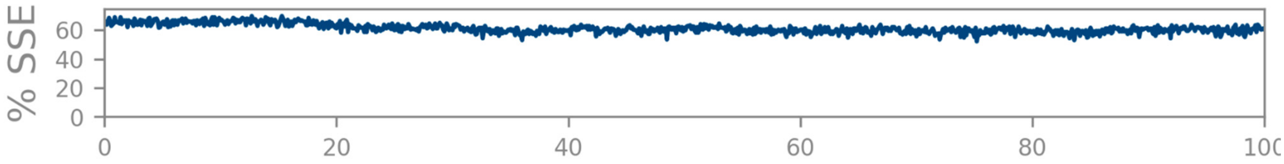

c)

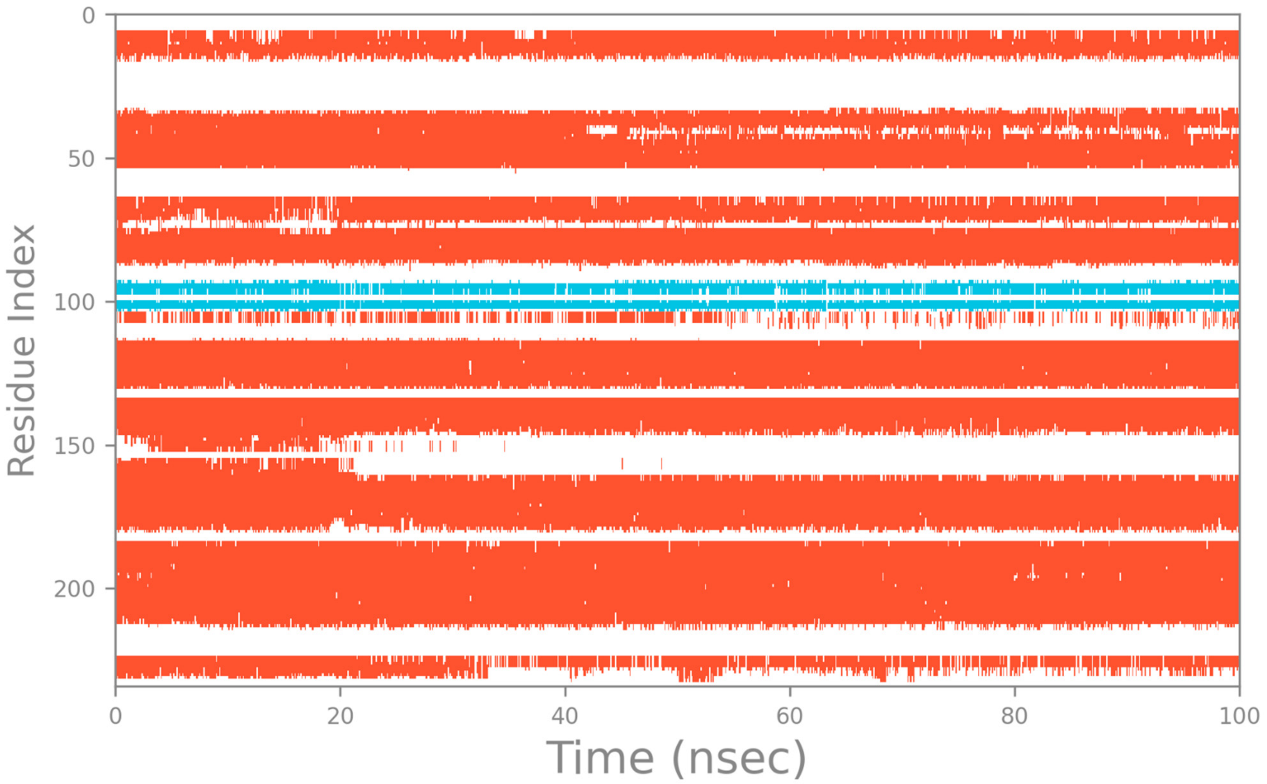

Figure S2. ER protein secondary structure elements (SSE) evaluation: **(a)** distribution of SSE by residue index across the entirety of the protein structure; **(b)** dynamic snapshot of structural changes over the simulation duration; **(c)** SSE assignment for each residue over time.

Figure S3a provides a 2D schematic vision of the ligand, color-coded to highlight the positions of rotatable bonds. Each rotatable bonds torsion is accompanied by a dial plot and corresponding bar plots (Figure S3b) of the same color. Dial plots depict the conformation of the torsion over the simulation, with the beginning centered in the plot and time evolution extending radially outwards. The accompanying bar plots present a summarized view of the torsion data, representing the probability density of the torsion at different angles. If torsional potential information is available, the plot includes the potential of the rotatable bond, expressed in kcal/mol on the left Y-axis.

a)

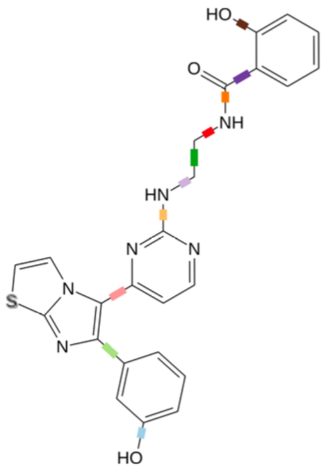

b)

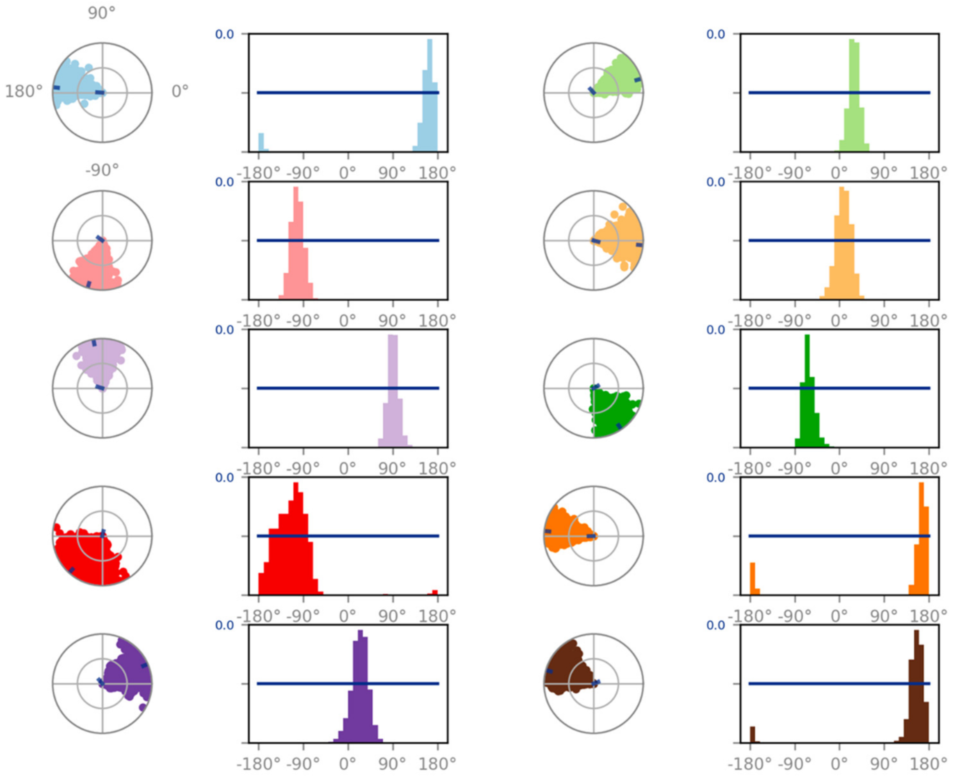

Figure S3. (a) 2D schematic representation of compound **755435**, color-coded to highlight the positions of rotatable bonds, each distinct color (blue, light green, pink, light orange, light purple, green, red, orange, purple, and brown) corresponds to specific rotable bonds within the molecule; **(b)** rotatable bond torsion dial plot and corresponding bar plots, when each color agrees to the correspondent in **(a)**.

Finally, in-depth structural analyses, consisting of various parameters, were conducted for each complex to elucidate key molecular characteristics.
Firstly, the Radius of Gyration (rGyr) was computed, providing insights into the 'extendedness' of the ligand. This calculation involved determining the RMS distance of each atom in the ligand from its center of mass, offering valuable information about the overall compactness or dispersion of the ligands conformation.
Simultaneously, the count of Intramolecular Hydrogen Bonds (intraHB) was determined, capturing the intricate network of hydrogen bonds formed within the ligand molecule. Furthermore, the Molecular Surface Area (MolSA) was calculated, quantifying the total surface area of the ligand in each complex. The Solvent Accessible Surface Area (SASA) was specifically determined to understand the portion of the ligands surface accessible to solvent molecules. Lastly, the Polar Surface Area (PSA) was computed to quantify the portion of the ligands surface consisting of polar atoms.
The detailed outcomes of these computations, presented in Figures S4-S5 for complexes **755435**/Hsp90 and **755435**/ER, respectively, collectively provide a nuanced and comparative perspective on the diverse molecular features of the ligand in different biological contexts.

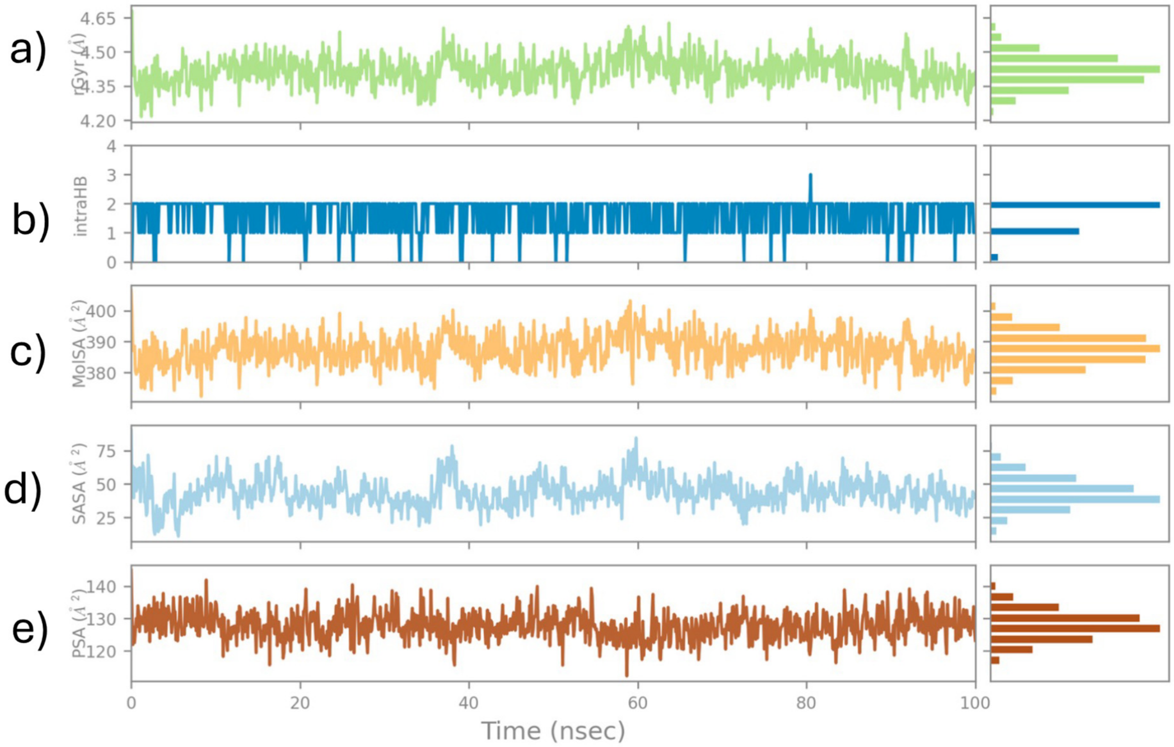

Figure S4. In-depth structural analyses for complex **755435**/Hsp90: **(a)** calculated rGyr over a 100ns simulation; **(b)** intraHB over 100ns; **(c)** MolSA variations over the 100ns simulation; **(d)** SASA changes during the 100ns simulation; **(e)** PSA dynamics over 100ns simulation.

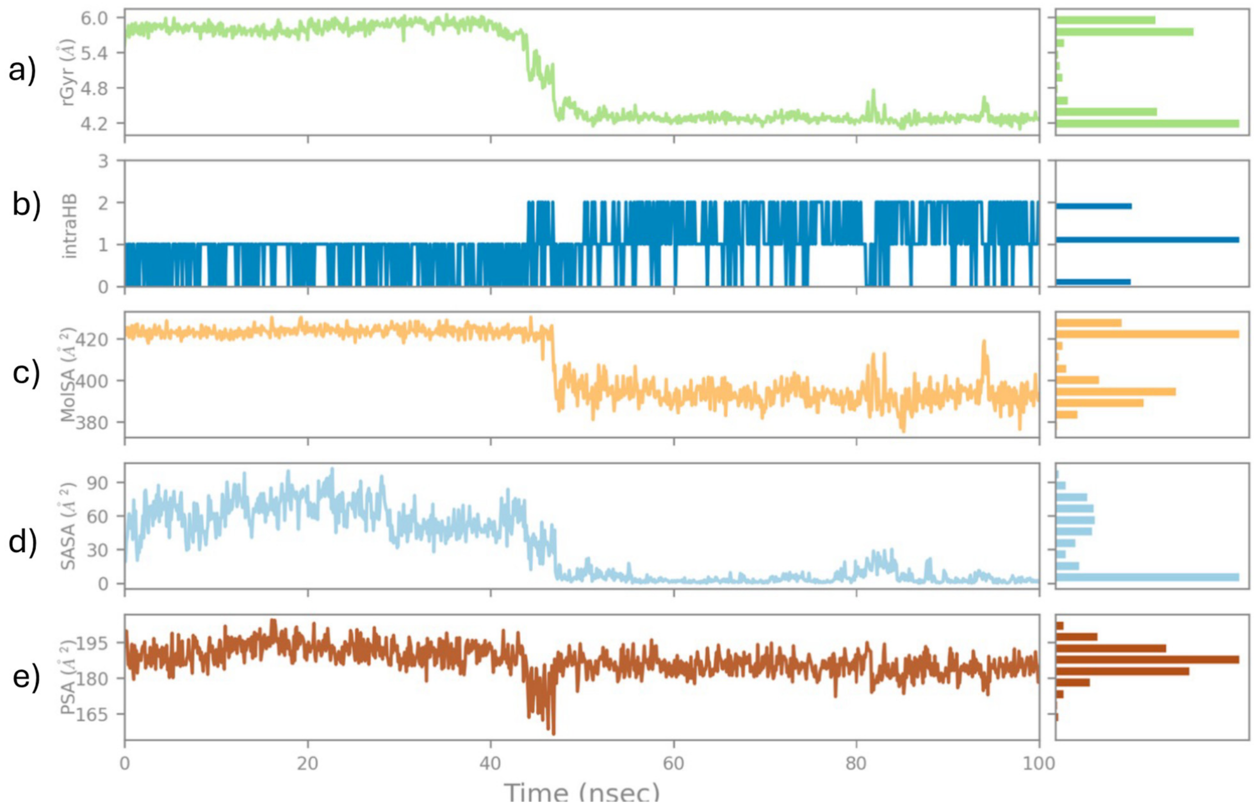

Figure S5. In-depth structural analyses for complex **755435**/ER: **(a)** calculated rGyr over a 100ns simulation; **(b)** intraHB over 100ns; **(c)** MolSA variations over the 100ns simulation; **(d)** SASA changes during the 100ns simulation; **(e)** PSA dynamics over 100ns simulation.

Figures S6, S7, and S8 present the P-RMSF plots for each protein—Hsp90 and ER—in complex with PF-04929113, UP-H64, and Raloxifene, respectively. These plots highlight regions of the proteins exhibiting the greatest fluctuations during the simulations. Notably, the peaks in the P-RMSF plots correspond to residues with higher flexibility, typically associated with loop regions or terminal segments, which are inherently more dynamic than the structured secondary elements, such as alpha helices and beta sheets, which generally display greater rigidity. In Figure S6, significant fluctuations exceeding 4.5 Å are observed around residue indices 50 and 200. These regions likely correspond to flexible segments of the protein, possibly located near solvent-exposed loops. Residues involved in ligand interactions, marked by green vertical bars, exhibit relatively low fluctuation values (RMSF < 1.5 Å). This indicates the structural rigidity of binding site regions, which is essential for maintaining stable ligand-protein interactions and ensuring the functional integrity of the complex. Figure S7 similarly reveals pronounced peaks in the P-RMSF plot around residue indices 25, 50, and 180, with fluctuations exceeding 3.5 Å. These peaks suggest regions of high mobility, potentially corresponding to loop or terminal domains. Residues critical for ligand interactions, again marked by green vertical bars, are predominantly located in areas with lower fluctuations (RMSF < 1.5 Å), underscoring their role in stabilizing the protein-ligand complex and maintaining structural integrity within the binding site. In Figure S8, significant peaks appear at residue indices 25 and 50, with fluctuations exceeding

2.5 Å. These findings are consistent with the dynamic nature of loop or terminal regions, while residues involved in ligand interactions display limited fluctuations, emphasizing the stability required for effective ligand binding. Overall, the comparative analysis of P-RMSF plots highlights the dynamic behavior of specific protein regions and underscores the structural rigidity of ligand-binding sites, aligning with their functional importance in maintaining stable protein-ligand complexes.

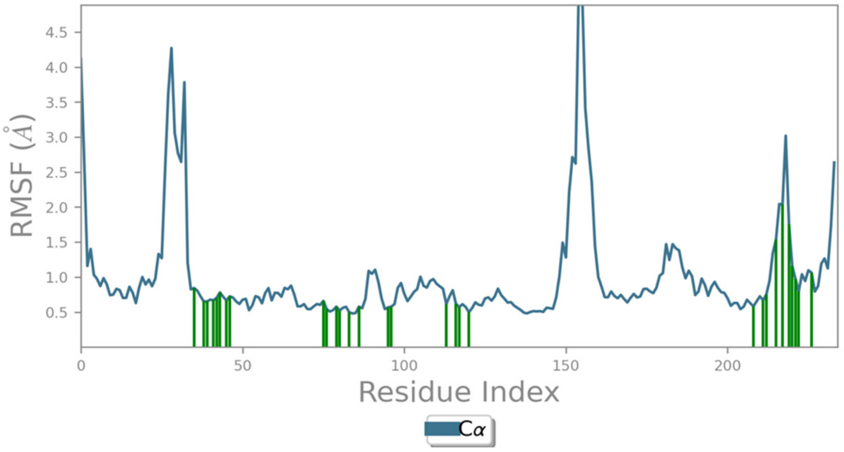

Figure S6: Calculated P-RMSF during the simulation for ER in complex with Raloxifene;

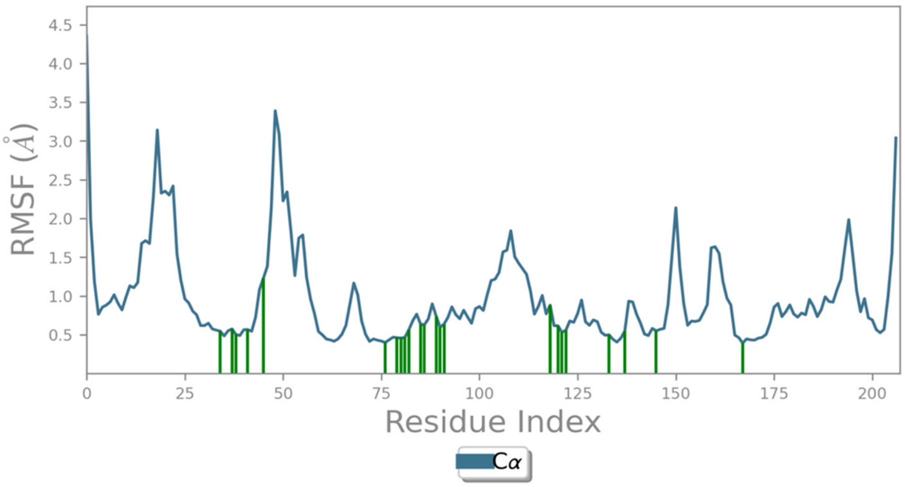

Figure S7: Calculated P-RMSF during the simulation for Hsp90 in complex with PF-04929113.

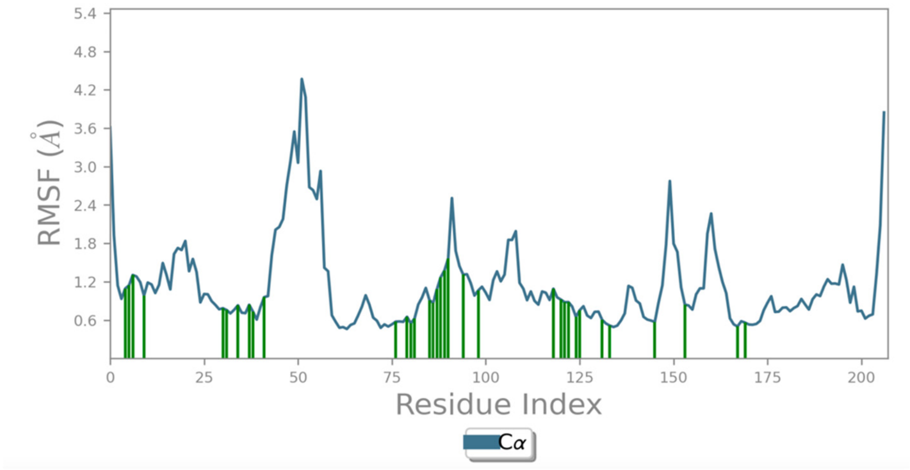

Figure S8: Calculated P-RMSF during the simulation for Hsp90 in complex with UP-H64.

The L-RMSF results, presented in Figures S9, S10, S11 for ER and Hsp90 respectively, provide a detailed view of the ligand's atomic fluctuations. In the figure S9, the RMSF profile for Raloxifene reveals low to moderate fluctuations, with peaks observed primarily at the termini of the ligand structure. These peaks indicate localized flexibility in specific functional groups, which may correspond to interactions with dynamic regions of the ER binding pocket. The overall RMSF values remain below 2 Å, indicating that Raloxifene maintains a stable interaction with ER throughout the simulation. In the figure S10, representing PF-04929113 bound to HSP90, demonstrates a distinct fluctuation pattern compared to the ER complex. The RMSF values are moderate, with a notable peak around residue index 14, suggesting significant flexibility in this region. This localized increase in atomic mobility could correspond to a functional group involved in transient interactions with residues of HSP90. Despite the presence of a high-entropy region, the overall RMSF values remain below 1.5 Å. In the figure S11, The RMSF profile of the ligand UP-H64 in complex with HSP90 shows pronounced atomic fluctuations, with values exceeding 4 Å in specific regions, particularly at the termini of the ligand structure. This pattern suggests high mobility of certain functional groups, which may engage in dynamic and transient interactions with the residues of the HSP90 binding pocket.

The fluctuation trend across the ligand indicates regions of significant conformational flexibility, pointing to potential structural entropy. However, some central regions of the ligand display lower RMSF values, indicating greater stability in these areas.

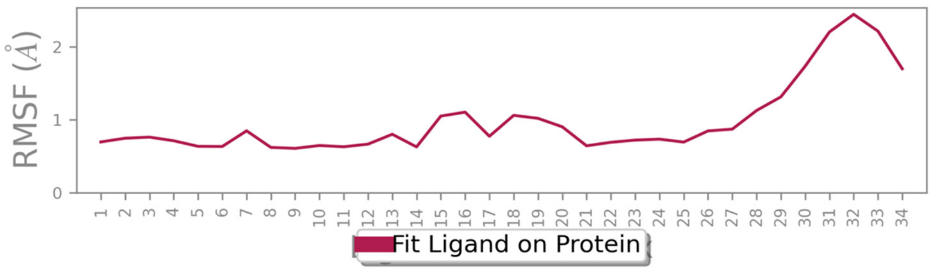

Figure S9: calculated L-RMSF during the simulation for Raloxifene in complex with ER;

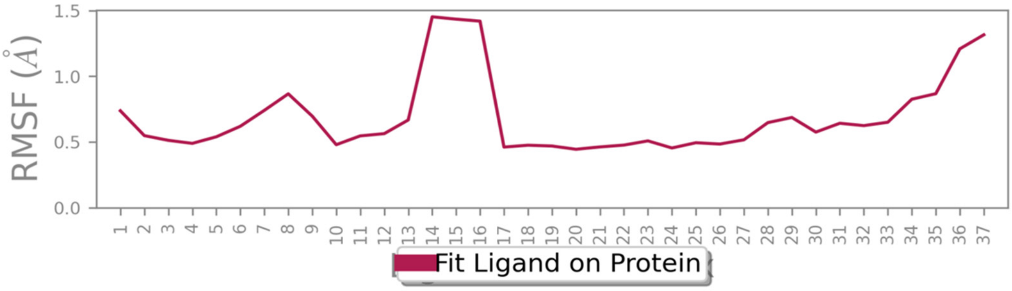

Figure S10: calculated L-RMSF during the simulation for PF-04929113 in complex with HSP90;

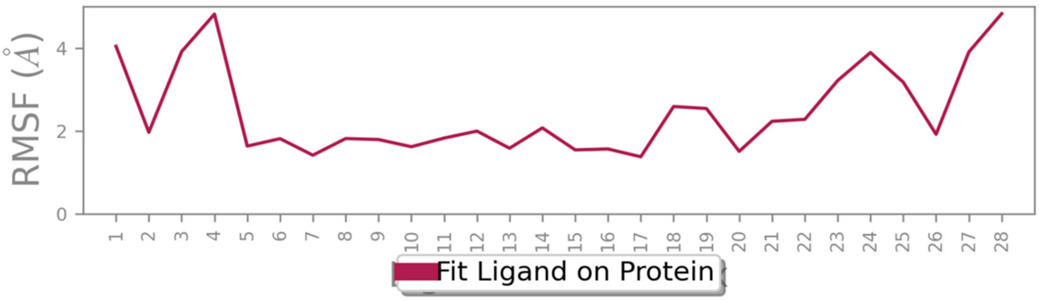

Figure S11: calculated L-RMSF during the simulation for UP-H64 in complex with HSP90;
